# A Biomineralization Strategy for “Net”‐Like Interconnected TiO_2_ Nanoparticles Conformably Covering Reduced Graphene Oxide with Reversible Interfacial Lithium Storage

**DOI:** 10.1002/advs.201500176

**Published:** 2015-08-25

**Authors:** Qiang Zhang, Yong Yan, Ge Chen

**Affiliations:** ^1^ Key Laboratory for Green Catalysis and SeparationCollege of Environmental & Energy EngineeringBeijing University of TechnologyPingle yuan 100100124BeijingP. R. China

**Keywords:** biomineralization strategies, interfacial lithium storage, lithium ion batteries, reduced graphene oxide, TiO_2_

## Abstract

A green and simple biomineralization‐inspired method to create “net”‐like interconnected TiO_2_ nanoparticles conformably covering reduced graphene oxide (RGO) with high loading density is reported. This method uses polyamine as both the biomineralization agent and linker to manipulate the nucleation, growth, and crystallization of TiO_2_ nanoparticles on the surface of graphene oxide. The obtained TiO_2_/RGO composites demonstrate sub‐10‐nm TiO_2_ nanoparticles with (001) facets, ultrathin thickness (10–12 nm), and a high surface area of 172 m^2^ g^−1^. When used as anode material for lithium ion batteries, the material displayed excellent rate capability and long cycle life; a capacity of 155 mAh g^−1^ is obtained after 50 cycles at the rate of 5C (1C = 168 mA g^−1^) and a specific capacity of 115 mAh g^−1^ is retained after 2000 cycles at the rate of 25C, which is much higher than that of mechanically mixed TiO_2_/graphene composites. Detailed discharge curve analysis reveals that the high rate and cycle performance are partly a result of the reversible interfacial lithium storage of materials, which might be attributed to the pores in the TiO_2_ nets on the RGO and may provide a sufficient number of interfaces for accepting both electrons and lithium ions.

## 1 Introduction

Reduced graphene oxide (RGO) has great electronic conductivity, high specific surface area, chemical stability, and structural flexibility, making it an exceptionally promising and versatile building block for the design of functional materials, and it is widely used to prepare various hybrid materials.^1^, ^2^, ^3^ In particular, TiO_2_/RGO composites have attracted great attention for these hybrid systems, leading to many promising applications in areas such as energy conversion and storage, photocatalysis, and sensing.^4^, ^5^, ^6^, ^7^, ^8^, ^9^, ^10^, ^11^, ^12^, ^13^, ^14^, ^15^ For example, integrating RGO with TiO_2_ leads to a much enhanced performance toward photocatalytic water splitting and dye degradation through the effective interfacial charge transfer;^4^, ^5^, ^6^ and the incorporation of RGO into the TiO_2_ matrix can improve electron transport while reducing charge recombination, thus enhancing the performance of dye‐sensitized solar cells;^7^, ^8^ also, the RGO/TiO_2_ electrode showed fast lithium storage in a lithium ion battery, indicating its potential application in electric vehicles;^9^, ^10^, ^11^ the RGO/TiO_2_ composites also exhibited interesting properties in supercapacitors,^12^ biosensors,^13^ photoinactivation of bacteria,^14^ and self‐cleaning applications.^15^ However, the lattice mismatch and poor affinity between RGO and TiO_2_ nanoparticles make it difficult to achieve in situ growth of well‐dispersed TiO_2_ nanoparticles on the surface of RGO, seriously hindering the application of TiO_2_/RGO; also, aggregation of nanoparticles often occurs in the synthetic process and may lead to poor device performance. Ensuring the nucleation and growth of TiO_2_ nanocrystals selectively on RGO instead of growth in solution is a challenge because of the poor lattice compatibility between RGO and TiO_2_. Also, the high TiO_2_ loading density often leads to the aggregation of nanoparticles on the RGO, which seriously hinders its performance. Using a delicate controlled sol–gel approach,^16^ space confinement method^17^ or linker/seed hydrothermal pathway,^18^ ultra‐dispersed TiO_2_ nanoparticles on graphene have been successfully synthesized; however, these reported methods are often complicated, which make them unsuitable for scalable production; in addition, the density of loaded particles is far from suitable.

Biomineralization‐inspired synthesis of TiO_2_ is a recently developed route that mimics the natural biosilicification process to carry out the nucleation, growth, and crystallization of TiO_2_ in ambient conditions.^19^, ^20^, ^21^, ^22^, ^23^, ^24^, ^25^, ^26^, ^27^, ^28^, ^29^, ^30^ Not only can the synthesis be carried out under environmentally benign conditions, it also provides a new pathway to tailor the physicochemical properties with significant advantages over conventional methods. Previous research has revealed that nitrogen‐containing organic molecules such as proteins,^19^, ^20^, ^21^, ^22^ peptides,^23^, ^24^ polypeptides,^25^ short‐chain polyamines,^26^ and synthetic polymers^27^, ^28^ can be used as biomineralization agents for fabricating various TiO_2_ nanostructures. Here, we report a biomineralization strategy toward the synthesis of a “net”‐like anatase TiO_2_ nanoparticle conformable covering on RGO. The resulting TiO_2_/RGO composites possess sub‐10‐nm anatase nanoparticles with high‐energy (001) facets, ultrathin thickness (10–12 nm), high‐density TiO_2_ loading (82 wt%), and a high surface area (172 m^2^ g^−1^). In particular, many TiO_2_ nanoparticles are interconnected by fused interfaces; thus, the voids between nanoparticles form unique pores on the RGO. Furthermore, the hybrid material exhibits excellent rate capability and cycle performance when used as the anode material in lithium ion batteries; a capacity of 155 mAh g^−1^ is obtained at the rate of 5C (1C = 168 mA g^−1^), and a capacity of 115 mAh g^−1^ is retained at 25C after 2000 cycles, which is much higher than that of mechanically mixed TiO_2_/graphene composites. Detailed discharge curve analysis reveals that the high rate and cycle performance is partly a result of the reversible interfacial lithium storage of materials, which might be attributed to the pores in the TiO_2_ “nets” on the RGO, which may provide a sufficient number of interfaces for accepting both electrons and lithium ions. This study demonstrates a good example of applying a biomineralization strategy toward inorganic materials synthesis with a combined perspective of improving electrochemical performance and using an eco‐efficient synthesis route.

## 2 Results and Discussion

**Figure 1:**
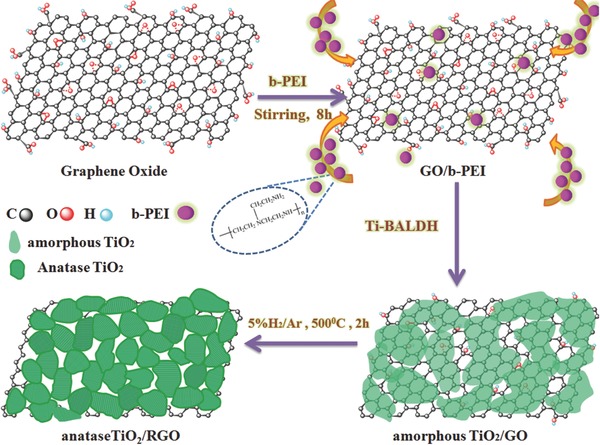
Schematic illustration of biomineralization strategy toward a single grain layer of TiO_2_ “nets” conformably covering reduced graphene oxide.

In this work, we use positively charged branched polyethylenimine (b‐PEI) as both biomineralization agent and linker (linking to GO) to realize the control of nucleation and subsequent growth of an amorphous TiO_2_ layer on the surface of GO. Unlike the traditional sol–gel approach, biomineralization‐inspired synthesis of TiO_2_ is often based on the active site of organic molecules catalyzing the hydrolysis of titanium precursors in aqueous solutions (the most commonly used titanium precursor is titanium(IV) bis(ammonium lactate) dihydroxide (Ti‐BALDH), which is stable under neutral aqueous solution), which means the subsequent titanium precursor condensation cannot happen when the active site is covered by the already formed amorphous TiO_2_ layer; because the biomineralization agent (b‐PEI) is linked to the GO, the nucleation and growth of TiO_2_ only occur on the surface of GO, thus effectively avoiding the free growth of TiO_2_ in solution. In a typical synthesis (**Figure** **1**), b‐PEI was adsorbed and accumulated on GO by electrostatic interaction; thus, the GO/b‐PEI can be used as a 2D catalytic template for promoting the hydrolysis of Ti‐BALDH under neutral pH conditions. This led to the selective formation of a thin layer of amorphous TiO_2_ deposited on graphene oxide instead of free growth in solution. After a thermal treatment process under an Ar (95%) + H_2_ (5%) atmosphere at 500 °C, the amorphous TiO_2_ layer is crystallized into anatase TiO_2_ accompanied by the reduction of GO, resulting in the formation of “net”‐like interconnected anatase TiO_2_ nanoparticles conformably covering the RGO. Due to the ambient condition, biomineralization inspired synthesis of TiO_2_ is often amorphous or partially crystallized, which is not suitable for many devices application;^19^, ^21^, ^22^, ^24^ thus, annealing is a simple method to further enhance the crystallinity of TiO_2_. Avoiding thermal processes (both annealing and solvothermal process) in the synthesis of high crystallized nano‐TiO_2_ is a challenge; to the authors' knowledge, the fabrication of crystalized nano‐TiO_2_/RGO nanocomposites without the use of thermal process is not reported yet. It is noteworthy that Kröger demonstrated the recombinant silaffins induced the formation high crystallized TiO_2_(rutile) micro‐sized crystals under ambient temperature and neutral pH;^20^ also, Chen reported a protein mediated high crystallized porous TiO_2_(rutile) by carrying out a slow dissolution‐recrystallization process under room temperature.^29^ The synthetic process is tracked by zeta potential measurement (Figure S1, Supporting Information); the GO demonstrates a potential of −23 ± 1 mV, corresponding to its negatively charged carboxyl group; the potential becomes +37 ± 2 mV after adsorbing positively charged b‐PEI; and the potential then changes reversibly to −18 ± 1 mV when amorphous TiO_2_ is deposited on the surface of GO, which is believed to come from the negatively charged surface of amorphous TiO_2_ in neutral aqueous solution.

**Figure 2:**
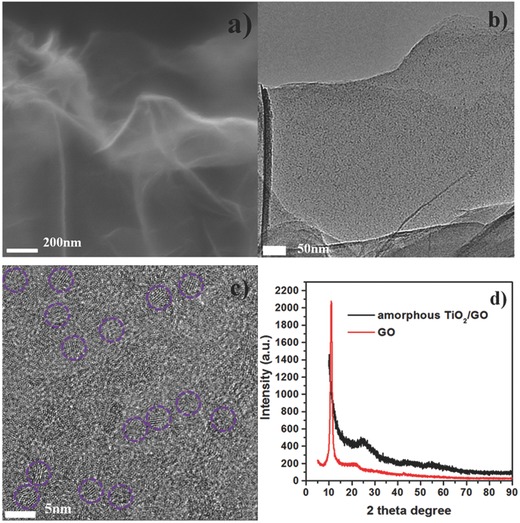
a) FESEM image of amorphous TiO_2_/GO, bar 200 nm; b) TEM image of amorphous TiO_2_/GO, bar 50 nm; c) HRTEM image of amorphous TiO_2_/GO, where the partial crystallized domains are marked by purple circles, bar 5 nm; d) XRD patterns of GO powder and amorphous TiO_2_/GO composite.

**Figure** **2**a shows a FESEM image of the amorphous TiO_2_/GO, in which many freestanding and ultrathin sheets can be observed; the thickness of a single amorphous TiO_2_/GO sheet is approximately 12–14 nm. TEM images also show the ultrathin sheet‐like nature, and amorphous TiO_2_ nanoparticles were observed on the GO sheets (Figure 2b). High‐resolution transmission electron microscopy (HRTEM) images reveal the very small crystal domain (approximately 2 nm) dispersed on the GO, indicating the partial crystallization of TiO_2_ (Figure 2c). The edge of graphene oxide (3–4 layers, indicated by the orange arrow) can be clearly observed in the image (Figure S2, Supporting Information), suggesting the ultrathin thickness of GO sheets in the amorphous TiO_2_/GO composites. The XRD pattern of the amorphous TiO_2_/GO composites exhibits a weak and broad diffraction peak centered at 2θ° = 26°, which could be attributed to the characteristic reflection of amorphous TiO_2_ (Figure 2d). The typical reflection at 2θ° = 11.3° of initial GO sheets disappeared, indicating the absence of layer‐by‐layer stacked GO sheets in the products.^30^ This result further confirms that GO sheets are separated by loading amorphous TiO_2_ nanoparticles uniformly.

**Figure 3:**
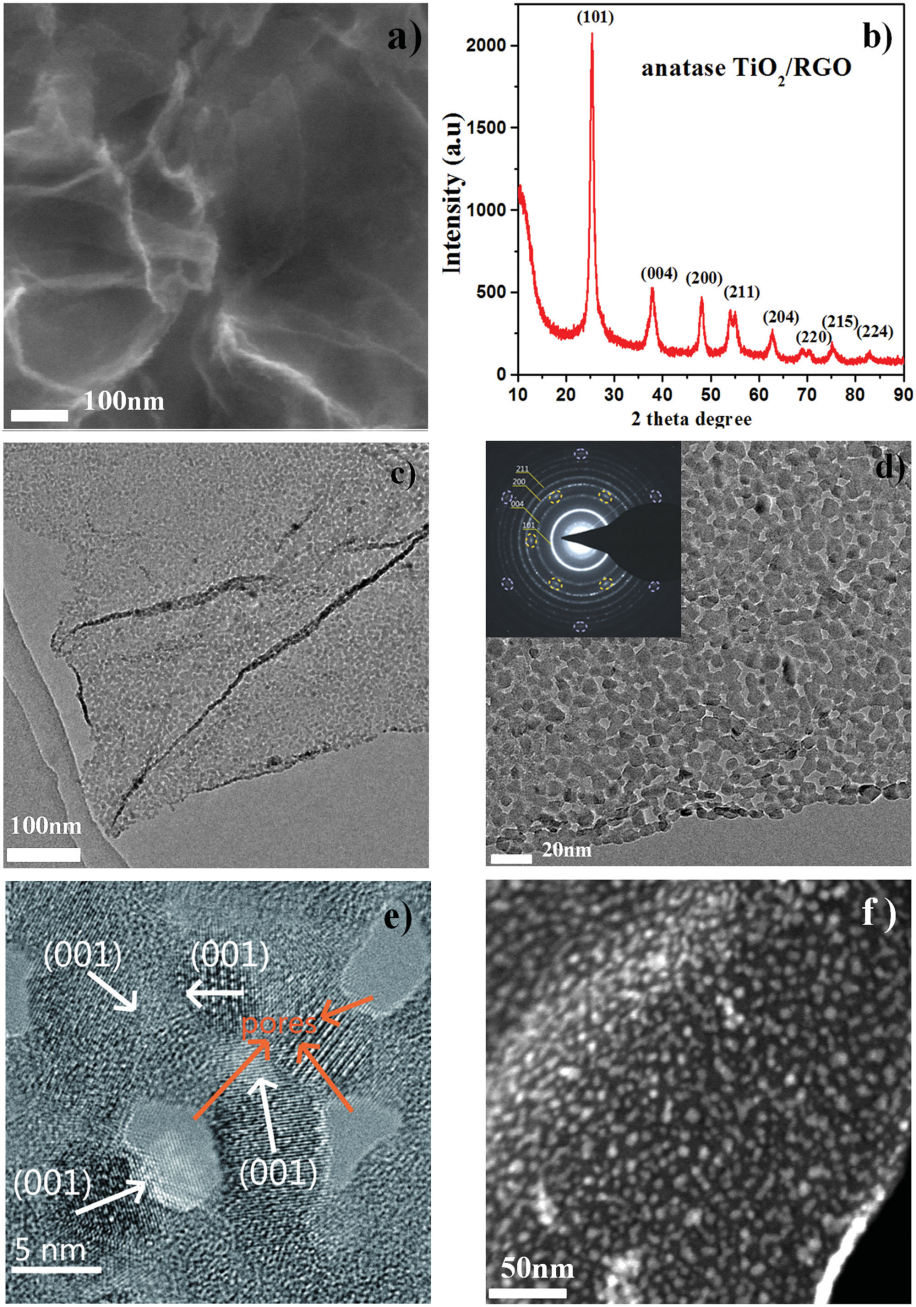
a) FESEM image of anatase TiO_2_/RGO composite, bar 100 nm; b) XRD pattern of anatase TiO_2_/RGO composite; c,d) TEM image of anatase TiO_2_/RGO composite under different magnifications; inset: SAED of anatase TiO_2_/RGO composite, where the spot marked by the circle indicates the existence of RGO; e) HRTEM image of anatase TiO_2_/RGO composite, where the white arrows indicate the direction of (001) facets; the orange arrows indicate the existence of pores on the composite, bar 5 nm; f) STEM–HAADF image of anatase TiO_2_/RGO composites, where the strip‐shaped part highlighted in the lower‐right corner indicates the thickness of the anatase TiO_2_/RGO composite, bar 50 nm.

After thermal treatment, FESEM of anatase TiO_2_/RGO sheets shows a similar morphology with the amorphous TiO_2_/GO, indicating that annealing did not change the morphology (**Figure** **3**a). No large particles are observed on the RGO, indicating the aggregation of nanoparticles could be effectively diminished by the thermal treatment. Figure 3b shows the XRD pattern of the TiO_2_/RGO composite, which matches the anatase phase (JCPDS 21‐1272) well.^31^ Using Scherer's formula to analyze the (101) facet, the average crystal size of TiO_2_ nanoparticles is determined to be approximately 9 nm. Because of the broad diffraction of the (101) facet of the obtained material, the (002) facet of RGO might be overlapped at a similar 2θ° location. However, no obvious diffraction peak at 2θ° = 26.3 is observed, suggesting the absence of stacked RGO.

Figure 3c shows the TEM characterization results for the anatase TiO_2_/RGO. A low‐resolution TEM image displays the typical sheet‐like morphology loaded with a large amount of small nanoparticles. A magnified TEM image reveals that the TiO_2_ nanoparticles are well dispersed on the RGO; in particular, many nanoparticles are interconnected by fused interfaces between nanoparticles (Figure 3d, Figure S3, Supporting Information). Thus, the voids between nanoparticles form pores on the composites. Compared with the reported porous TiO_2_/RGO, this structure is different from the pores inside the nanoparticles or bulk mesoporous TiO_2_;^10^, ^11^, ^18^ it is more like a “net” of interconnected TiO_2_ nanoparticles conformably covering the RGO. Such a novel and unique dispersion of TiO_2_ nanoparticles on RGO could be attributed to the biomineralization‐inspired synthetic pathway; note the GO could be replaced by other nanocarbons such as carbon nanotubes and carbon spheres, and uniformly dispersed TiO_2_ nanoparticles could be deposited on carbon nanotubes or carbon spheres (Figure S4, Supporting Information). The results suggest that the demonstrated biomineralization strategy could be expanded to prepare other inorganic material/carbon composites. The HRTEM image shows crystalline TiO_2_ nanoparticles on RGO; in addition to the commonly observed d spacing of 0.35 nm corresponding to the (101) facets of anatase TiO_2_, a well‐defined crystalline lattice can be identified with a d spacing of 0.23 nm, corresponding to the (001) facet of anatase TiO_2_ (Figure 3e). This observation is interesting because the surface energy of the (001) facet (0.90 J m^−2^) is higher than that of the (101) facet (0.44 J m^−2^); thus, anatase nanoparticles are usually observed in a truncated bipyramidal shape dominated by (101) facets.^32^, ^33^ Although some TiO_2_/RGO composites with anatase (001) facets have been successfully fabricated, a corrosive chemical agent (hydrofluoric acid) is often used to obtain high‐energy facets because it can reduce the surface energy to promote isotropic growth.^34^, ^35^, ^36^ It has been reported that nanostructured TiO_2_ with (001) facets showed superior electrode performance and photocatalytic properties;^37^, ^38^ thus, the “green” synthesis of nano‐TiO_2_ with controlled facets is challenging and highly desirable. In our work, the formation of (001) facets of anatase TiO_2_ is believed to come from the unique synthetic pathway, which might provide a new approach to tailor the facets of TiO_2_. Furthermore, a STEM‐HAADF image clearly confirms the well‐dispersed nanoparticles on the RGO (Figure 3f); the strip‐shaped part highlighted in the lower‐right corner of the image might be caused by the partial curl of the sheet, indicating the ultrathin thickness (about 10–12 nm) of the anatase TiO_2_/RGO composite. This result agrees well with the FESEM characterization of anatase TiO_2_/RGO composites. Furthermore, an energy‐dispersive X‐ray spectroscopy (EDS) line scan analysis demonstrates the distribution of C, N, O, and Ti species; while the Ti and O species are dispersed on the particles, the C and N species are dispersed along the line (Figure S5, Supporting Information). The N species might be attributed to pyrolyzed b‐PEI. Also, the elemental EDS mapping analysis confirms the existence of C, N, O, and Ti species, which are uniformly dispersed on the composites (Figure S6, Supporting Information). The selected‐area electron diffraction (SAED) pattern shows two series of well‐defined diffraction patterns (Figure 3d, inset), which can be assigned to RGO (marked with purple and orange circles) and anatase TiO_2_, respectively, indicating the effective TiO_2_ crystallization and reduction of graphene. The SAED pattern of anatase TiO_2_ contains a series of well‐defined Debye–Scherrer rings, confirming its polycrystalline nature (Figure 3d, inset). The calculated d spacing values of 3.5, 2.3, 1.9, and 1.6 Å correspond to the miller indices (101), (004), (200), and (211) facets of anatase.

**Figure 4:**
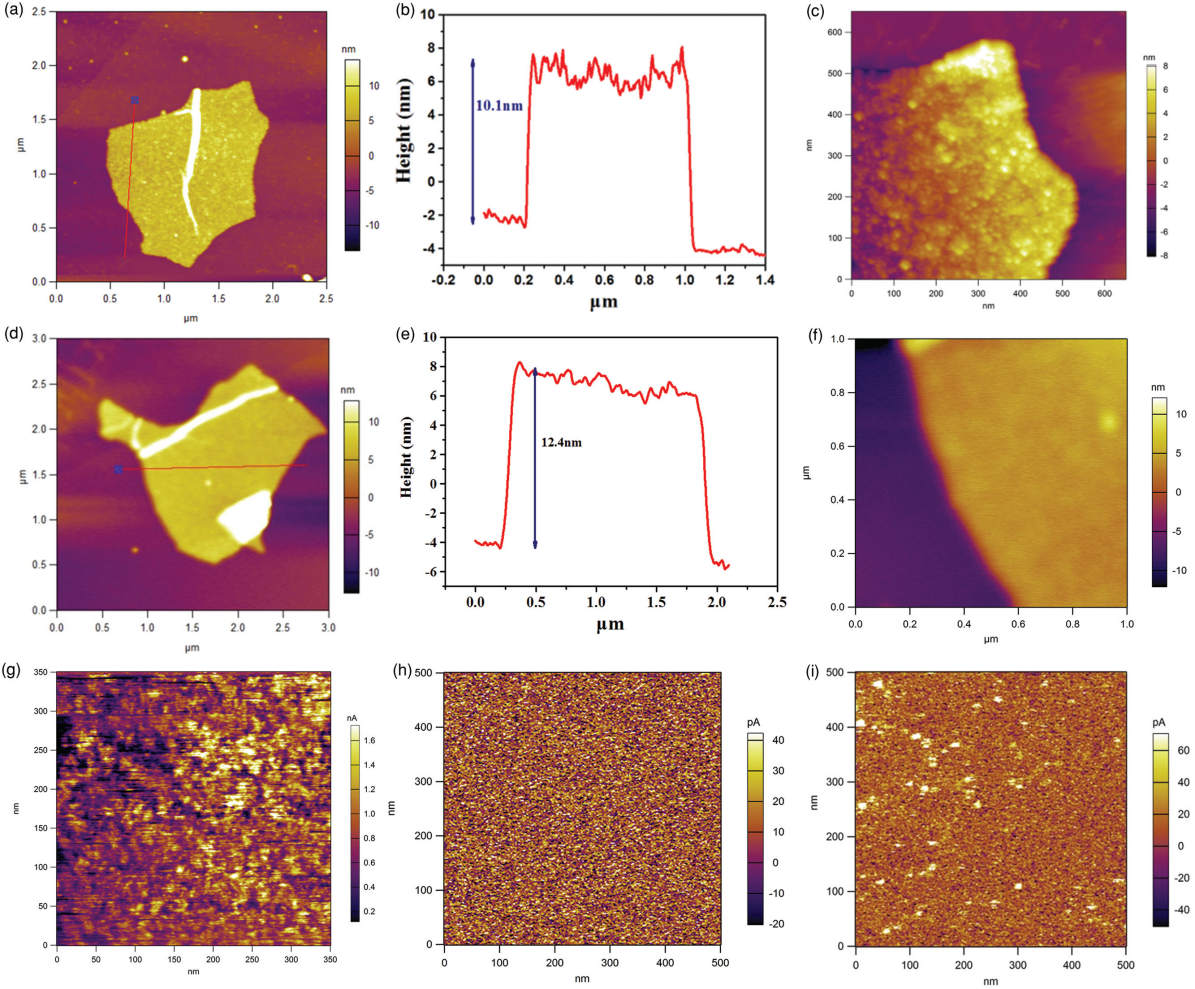
a) Topography image of anatase TiO_2_/RGO on the highly oriented pyrolytic graphite (HOPG) substrate, b) the corresponding height‐profile analysis along the line in a), and c) the enlarged topography image of anatase TiO_2_/RGO. d) Topography image of amorphous TiO_2_/GO on the HOPG substrate, e) the corresponding height‐profile analysis along the line in d), and d) the enlarged topography image of amorphous TiO_2_/GO. g) Current image of anatase TiO_2_/RGO under a bias of 3 V; h) current image of amorphous TiO_2_/GO under a bias of 3 V; i) current image of amorphous TiO_2_/GO under a bias of 5 V.

AFM images are shown in **Figure** **4**. Topography surface scans (tapping mode) for single anatase TiO_2_/RGO and amorphous TiO_2_/GO composites are shown in Figure 4a and 4d, respectively, which reveal the same morphology and structure as shown in the earlier SEM and TEM results. The anatase TiO_2_/RGO composite displays 2D features with a thickness of 10.0 nm (Figure 4b), while the amorphous TiO_2_/GO composite exhibits a thickness of 12.4 nm (Figure 4e); both are much thicker than graphene oxide (approximately 1.5 nm) (Figure S7, Supporting Information). The line of anatase TiO_2_/RGO shows more oscillation than that of the amorphous TiO_2_/GO composite, which might be attributed to the “net”‐like TiO_2_ nanoparticles on the surface of RGO; the amorphous TiO_2_ layer on GO is more smooth, leading to less oscillation in the line analysis. The result agrees well with the data given above for the TEM and SEM measurements. To further examine the electron transport properties of the obtained anatase TiO_2_/RGO composites, conductive‐AFM (ORCA mode) was used to probe the nanoscale electronic properties.^39^, ^40^ In the current imaging mode, a conductive tip scans over the single RGO/TiO_2_ composite surface to collect charge carriers at a certain fixed applied bias and the nanoscale current structure of the selected scan region is mapped out in the dark condition. Figure 4g–i show the dark current image of anatase TiO_2_/RGO and amorphous TiO_2_/GO composites, respectively; the selected scan areas are from the single anatase TiO_2_/RGO and amorphous TiO_2_/GO composites shown in Figures S8 and S9 (Supporting Information) (the selected area is indicated by the black square). A higher current can be seen in the selected region of the anatase TiO_2_/RGO composite; the current range of anatase TiO_2_/RGO is from hundreds of pA to several nA under a bias of 3 V. However, there is almost no current in the scanned area of the amorphous TiO_2_/GO composite under the same bias (3 V), and a very small current (tens of pA) is obtained under a higher bias (5 V) for the amorphous TiO_2_/GO composite. The results suggest a more efficient electron transport for the anatase TiO_2_/RGO composite, which might be useful in the electrochemical applications.

Raman spectroscopy is a powerful approach to characterize the ordered/disordered structure of RGO; in the Raman spectrum (Figure S10, Supporting Information), a D band at 1350 cm^−1^ is assigned to the edge planes and disordered structure, and a G band at 1580 cm^−1^ is assigned to the vibration of sp^2^‐bonded carbon atoms in a 2D hexagonal lattice. Also, the Raman spectrum indicates the characteristic peaks of anatase TiO_2_ at 147 cm^−1^.^31^ TGA of TiO_2_/RGO further demonstrates that the weight content of RGO in the composite is approximately 18% (Figure S11, Supporting Information), and the loading density of TiO_2_ (82 wt%) is higher than that of other reported ultra‐dispersed TiO_2_/RGO composites.^16^, ^17^, ^18^

N_2_ adsorption/desorption isotherms were used to investigate the surface areas and pore structures of materials (Figure S12, Supporting Information). The Brunauer‐Emmett‐Teller (BET) specific surface areas of amorphous TiO_2_/GO and anatase TiO_2_/RGO are 185 and 172 m^2^ g^−1^, respectively; both isotherms show type III curves with distinct hysteresis loops, which are different from the previously reported type IV curves for porous TiO_2_/RGO composites.^10^, ^11^, ^18^ After the thermal treatment, the densification of amorphous TiO_2_ and grain growth is believed to lead to a decrease in surface area; however, the formation of voids between particles after thermal treatment might improve the surface area. Thus, only a very slight decrease in BET surface area is observed after thermal treatment. Barrett‐Joiner‐Halenda (BJH) results suggest the sizes of pores range from 2 to 5 nm (Figure S12, Supporting Information). For use as an electrode or photocatalyst, such a porous structure and high surface area may provide more active sites and facilitate charge carrier transport, resulting in an improvement of the electrochemical or photocatalytic performance.

**Figure 5:**
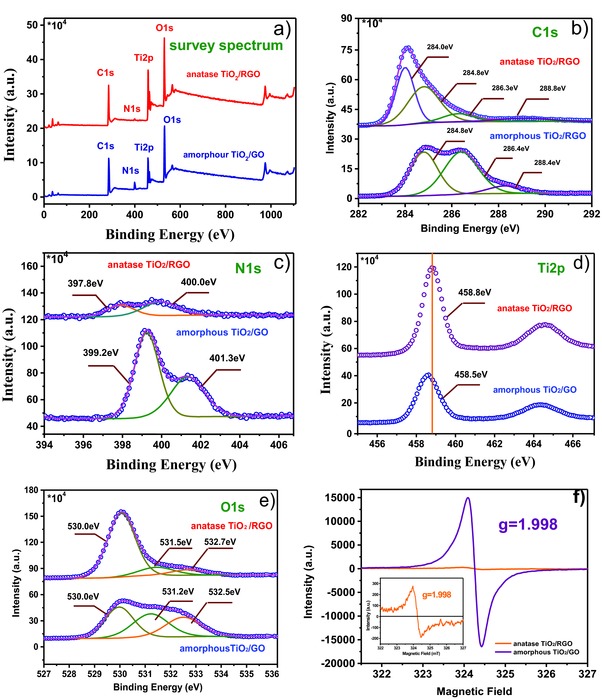
a) Survey XPS; b) C 1s; c) N 1s; d) Ti 2p; and e) O 1s spectra of anatase TiO_2_/RGO and amorphous TiO_2_/GO composites. f) EPR spectra of anatase TiO_2_/RGO and amorphous TiO_2_/GO composites, inset: enlargement of EPR spectra of anatase TiO_2_/RGO.

XPS was used to characterize the chemical environment of the elements in the amorphous TiO_2_/GO and anatase TiO_2_/RGO composites. **Figure** **5**a depicts the full spectra of both samples, in which C, Ti, O, and N species are observed. The C 1s spectra of both samples are shown in Figure 5b; three typical peaks can be observed for amorphous TiO_2_/GO, corresponding to chemically different species located at 284.8, 286.4, and 288.4 eV, respectively. The peak at 284.8 eV is believed to originate from sp^2^‐hybridized carbon from GO or aliphatic CH–CH in adventitious carbon; the peak at 286.4 eV is attributed to nitrogen‐ and oxygen‐bound species such as C–N, C–O, and C–OH; and the peak at 288.2 eV is assigned to carboxylate (O=C–O).^41^, ^42^ For anatase TiO_2_/RGO composites, the above‐mentioned three peaks are also observed; however, the intensities of the latter two peaks (at 286.3 eV and 288.8 eV) are much smaller, indicating the removal of most N‐ and O‐containing groups after thermal treatment. Interestingly, an additional C 1s peak with high content emerges at 284.0 eV, which is not observed in other anatase TiO_2_/RGO reports; the binding energy of this peak is much higher than the titanium carbide (Ti^4+^‐C) peak at 281.5 eV,^43^ and it could be assigned to carbon from RGO bonded to the anatase TiO_2_. A similar peak is observed for chemically bonded TiO_2_‐B/RGO.^44^ This result suggests a strong interaction between anatase TiO_2_ and RGO. The N 1s spectra are given in Figure 5c; two peaks are observed for amorphous TiO_2_/GO, at 399.2 and 401.3 eV. The former is believed to come from the amino groups (–NH_2_) of b‐PEI, and the latter is attributed to the protonated amino groups (–NH_3_^+^) of b‐PEI.^45^ For anatase TiO_2_/RGO, two peaks centered at 397.8 and 400.0 eV are observed; the peak at 397.8 eV is assigned to pyridinic N from nitrogen‐doped carbon, which might originate from pyrolyzed b‐PEI; the peak at 400.0 eV might be attributed to two possible sources: one is the overlap of quaternary N and pyridonic N (or pyrrolic N) from nitrogen‐doped carbon,^46^ the other is interstitial nitrogen bound to the lattice oxygen of TiO_2_.^47^ The Ti 2p spectrum of anatase TiO_2_/RGO contains peaks at 458.8 and 464.6 eV (Figure 5d), which are at slightly higher binding energies than those of amorphous TiO_2_/GO composites (≈458.5 and ≈464.3 eV). These peaks might appear at higher energy for anatase TiO_2_/RGO because of the stronger interaction between TiO_2_ and RGO, which indicates that the anatase TiO_2_ nanoparticles are strongly coupled to the RGO.

**Figure 6:**
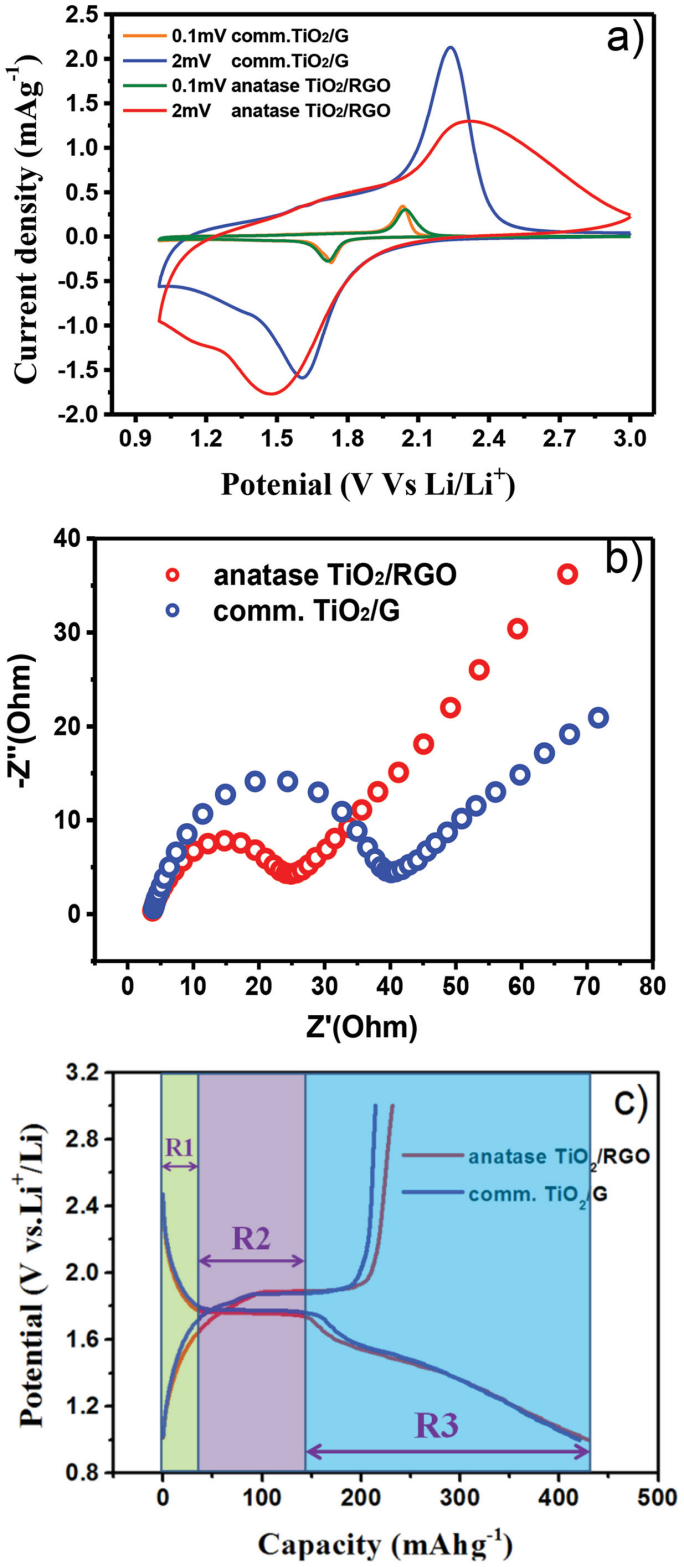
a) CVs for anatase TiO_2_/RGO and comm.TiO_2_/G electrodes measured at a scan rate of 0.1 and 2 mV s^−1^, respectively. b) Nyquist plots for anatase TiO_2_/RGO and comm.TiO_2_/G. c) Initial galvanostatic discharge/charge curves for anatase TiO_2_/RGO and comm.TiO_2_/G electrodes at a rate of 0.2C in the potential window of 1.0–3.0 V; the R1, R2, and R3 regions indicated by the different colors show the capacities of the three regions, which are based on the initial discharge curve of anatase TiO_2_/RGO in Figure 6c.

The O 1s spectra are also illustrated in Figure 5e. Three peaks are observed, at 530.0, 531.2, and 532.5 eV; the binding energy at 530.0 eV could be ascribed to Ti–O–Ti (lattice O), the peak at 531.2 eV is believed to originate from Ti–O–H, and the peak at 532.5 eV could be assigned to C–O–H.^22^ The contents of the latter two species decreased greatly after thermal treatment, confirming the removal of a large amount of oxygen‐containing groups in the amorphous TiO_2_/GO composite. EPR spectroscopy is often used to characterize the formation of defects (such as Ti^3+^ species and O^2−^ or oxygen vacancies) in TiO_2_, which greatly influence its physical and chemical properties.^48^, ^49^ A strong response at a *g* value of 1.998 was observed for amorphous TiO_2_/GO composites (Figure 5f); it is well known that the EPR signal of Ti^3+^ defects and O^2−^ and oxygen vacancies is located at *g* = 1.960–1.990, 2.020, and 2.003, respectively.^50^, ^51^, ^52^ Thus, this signal cannot be classified as a Ti^3+^ defect or an O^2−^ or oxygen vacancy, indicating the formation of a new defect species. According to the line shape (symmetry and line width) of the signal, the signal might be attributed to the existence of carbon‐centered radicals coming from the strong interaction between the biomineralization agent (b‐PEI) and amorphous TiO_2_. Wang reported a strong symmetrical EPR signal at *g* = 1.998 in a titanium‐defected undoped anatase TiO_2_, which is similar to the EPR result obtained in our work.^53^ After annealing, this signal intensity decreases greatly. One explanation might be that the b‐PEI was pyrolyzed to nitrogen‐doped carbon and the amorphous TiO_2_ was crystallized to anatase TiO_2_. The presence of defects in anatase TiO_2_/RGO indicated by these signal likely results in the formation of Ti–O–C centers, suggesting a strong interaction between anatase TiO_2_ and RGO. The lithium‐storage performance of anatase TiO_2_/RGO was evaluated; for comparison, the performance of commercial 12‐nm‐sized anatase TiO_2_ nanoparticles (comm.TiO_2_, surface area 178 m^2^ g^−1^ purchased from Alfa Aesar) mechanically mixed with graphene (G) nanopowder (flakes, approximately 12 nm, purchased from Graphene Supermarket, USA) was also tested under the same conditions. **Figure** **6**a shows the CV curves of TiO_2_/RGO and comm.TiO_2_/G electrodes measured at scan rates of 0.1 and 2 mV s^−1^. At a scan rate of 0.1 mV s^−1^, the CV curves of both TiO_2_/RGO and comm.TiO_2_/G show a pair of cathodic/anodic peaks at 1.7 and 2.0 V, respectively, corresponding to the characteristic lithium ion insertion/extraction potentials for the anatase TiO_2_. At a scan rate of 2 mV s^−1^, an obvious shoulder peak was observed at 1.1 V for the TiO_2_/RGO electrode, which might be attributed to lithium storage occurring at nanoparticle surfaces/interfaces; this shoulder peak is much less obvious for the comm.TiO_2_/G electrode. The Nyquist plots for anatase TiO_2_/RGO and comm.TiO_2_/G in Figure 6b display a single semicircle in the high‐frequency region and a sloping straight line in the low‐frequency range, corresponding to the charge transfer resistance (*R*_ct_) and solid‐state diffusion of lithium (*Z*_w_), respectively. The smaller semicircle diameter for the anatase TiO_2_/RGO electrode indicates a smaller charge transfer resistance compared with the comm.TiO_2_/G electrodes, indicating that the well‐dispersed TiO_2_ on RGO facilitates rapid charge transfer. This result suggests that TiO_2_/RGO may possess better electrochemical performance for lithium ion batteries than comm.TiO_2_/G.

Figure 6c shows the initial galvanostatic discharge/charge curves for anatase TiO_2_/RGO and comm.TiO_2_/G conducted in the potential window of 1.0–3.0 V at a rate of 0.2C (1C = 168 mA g^−1^). A high initial discharge capacity of 430 mAh g^−1^ and charge capacity of 231 mAh g^−1^ were obtained at 0.2C for anatase TiO_2_/RGO, corresponding to an initial Coulombic efficiency of 53.7%; comm.TiO_2_/G demonstrated a similar initial discharge capacity but smaller charge capacity of 214 mAh g^−1^, corresponding to an initial Coulombic efficiency of 50.8%. Because of surface reactions with the electrolyte upon reduction, the irreversible capacity loss in the first cycle is commonly observed for many high surface areas of nanostructured TiO_2_.^54^, ^55^, ^56^, ^57^, ^58^ According to the literature, the discharge curves of anatase TiO_2_ can be divided into three consecutive voltage regions;^59^, ^60^, ^61^, ^62^ first, the voltage decreased rapidly from 3.0 to about 1.75 V (vs. Li^+^/Li), corresponding to the formation of a solid solution domain (region 1). In the second region, a plateau is observed at a voltage of 1.75 V, displaying a biphasic region where anatase TiO_2_ is expected to coexist with Li‐rich phases (Li*_x_*TiO_2_) (region 2), and the maximum *x* is 0.5 for a fully reversible reaction, accompanied by a phase transformation from tetragonal TiO_2_ to orthorhombic Li_0.5_TiO_2_. The third region is from 1.75 to 1.0 V, where the voltage decreases linearly with increasing capacity, suggesting further lithium storage occurred at nanoparticle surfaces/interfaces (region 3). Provided the electrode material is made of a Li‐accepting phase and an electron‐accepting phase, the interfacial lithium storage could be generated at the interface of nanosized materials. This kind of Li storage was first proposed by Jamnik and Maier in 2003, and is named the “job‐sharing” mechanism.^63^, ^64^, ^65^ Because of its fast Faradaic reactions at the surface and long‐term cycling stability, the development of interfacial lithium storage is of great interest for high power density applications.^66^, ^67^, ^68^, ^69^, ^70^

**Figure 7:**
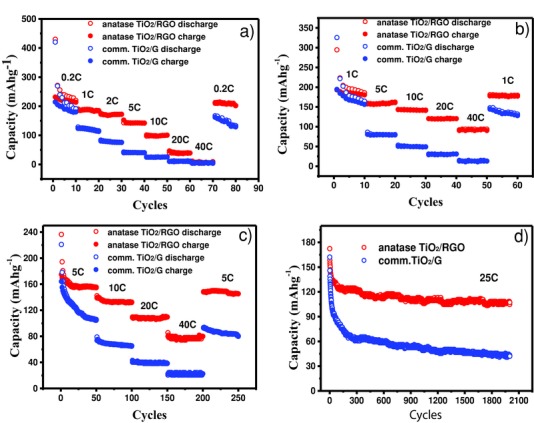
Rate performance of anatase TiO_2_/RGO and comm.TiO_2_/G electrodes at charging/discharging rates between a) 0.2C and 40C, b) 1C and 40C, and c) 5C and 40C. d) Cycling performance of anatase TiO_2_/RGO and comm.TiO_2_/G electrodes at a high rate of 25C for 2000 cycles.

**Figure** **7**a–c display the rate performances of anatase TiO_2_/RGO and comm.TiO_2_/G at various starting rates (0.2C, 1C, and 5C). For the starting rate of 0.2C, the capacities of anatase TiO_2_/RGO are approximately 220, 185, 172, 142, 100, 39, and 9 mAh g^−1^ at step rates of 0.2, 1, 2, 5, 10, 20, and 40C, respectively, while comm.TiO_2_/G shows values of 191, 115, 76, 40, 25, 10, and 5 mAh g^−1^ at the corresponding rates. It can be observed that the capacities of anatase TiO_2_/RGO are higher than those of comm.TiO_2_/G at all rates; however, the capacities of anatase TiO_2_/RGO decrease greatly when the step rates are above 10C. For the starting rate of 1C, anatase TiO_2_/RGO displays an excellent rate performance of 162, 141, 120, and 93 mAh g^−1^ at step rates of 5, 10, 20, and 40C; the capacities at high step rates (10C, 20C, and 40C) are much higher than those starting from 0.2C; similar phenomena were also observed with the starting rate of 5C for anatase TiO_2_/RGO; in addition, a capacity of 155 mAh g^−1^ is retained at 5C after 50 cycles, indicating high reversibility. Shin suggested that low capacities at high step rates might be caused by irreversible lithium insertion occurring in region 3 at a low starting rate (0.2C), leading to lower capacities in the subsequent cycles at higher rates.^69^ When the material was initially measured at a high starting rate (1C and 5C), the lithium storage in region 3 is believed to be reversible, resulting in higher capacities in the following cycles at higher rates. Even at a high rate of 25C, a capacity of 125 mAh g^−1^ can still be delivered after 2000 cycles for TiO_2_/RGO composites, whereas the remaining capacity of comm.TiO_2_/G is only 80 mAh g^−1^ under similar conditions after 100 cycles; a capacity of only 43 mAh g^−1^ is retained after 2000 cycles (Figure 7d). Such LIBs performance is comparable with the existed state‐of‐art TiO_2_/RGO electrode materials (Table S1, Supporting Information).^9^, ^10^, ^11^, ^16^, ^18^, ^71^, ^72^, ^73^

**Figure 8:**
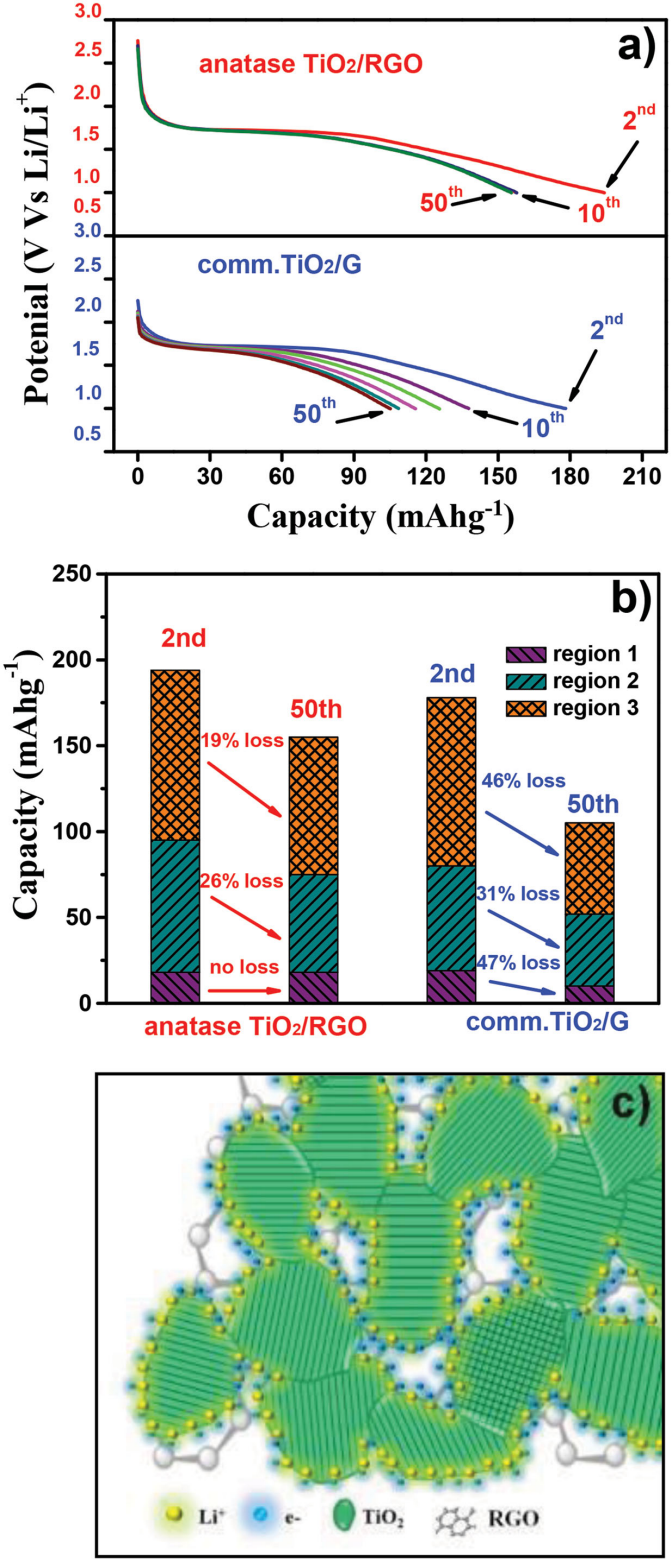
a) The 2nd, 10th, 20th, 30th, 40th, and 50th discharge curves for anatase TiO_2/_RGO and comm.TiO_2_/G electrodes at the rate of 5C. b) The regions 1, 2, and 3 capacity contribution analysis of anatase TiO_2_/RGO and comm.TiO_2_/G electrodes at the 2nd and 50th discharge curves, respectively. c) Schematic representation of interfacial lithium storage on the anatase TiO_2_/RGO composites.

To understand the remarkable rate performance and long cycle life of anatase TiO_2_/RGO composites, detailed capacity contributions for each region in the discharge curves at 5C were analyzed for both anatase TiO_2_/RGO and comm.TiO_2_/G. Considering the large side reaction of the initial discharge process, we chose the second‐ cycle discharge curve and the 50th cycle discharge curve of both samples in **Figure** **8**a for analysis. Figure 8b shows the three region contributions for the selected discharge curves of anatase TiO_2_/RGO; the capacity contributions are 18, 77, and 99 mAh g^−1^ at the second discharge curve for region 1, region 2, and region 3, respectively, accounting for 9.3%, 39.7%, and 51.0% of the total capacity, respectively. The capacity loss of region 3 is 19.1% after 48 cycles, while the capacity loss is 25.9% for region 2; no obvious capacity loss was observed for region 1. Additionally, the discharge curves in Figure 8a demonstrate that there is no obvious decrease in capacity for anatase TiO_2_/RGO from the 10th cycle to the 50th cycle, indicating the high reversibility. As for the comm.TiO_2_/G, the capacity contributions are 18, 61, and 98 mAh g^−1^ for region 1, region 2, and region 3 at the second discharge curves, respectively, accounting for 10.6%, 34.3%, and 55.1% of the total capacity, respectively. The capacity contribution and ratios of the three regions are similar to those of anatase TiO_2_/RGO; however, the capacity losses are 47.3%, 31.1%, and 45.9% after 48 cycles for region 1, region 2, and region 3, respectively (Figure 8b), which is much higher than those of anatase TiO_2_/RGO. The discharge curves in Figure 8a display the continued decrease in capacity from the second cycle to the 50th cycle. Because of the high ratio of the region 3 capacity contribution to the total capacity (>50%) in both samples, the dramatic loss in region 3 capacity (45.9%) might be the major factor for the poor cycle performance of comm.TiO_2_/G. The electrochemical performance comparison between anatase TiO_2_/RGO and comm.TiO_2_/G suggests that the high rate capability and good reversibility for anatase TiO_2_/RGO might be attributed to the reversible interfacial lithium storage (region 3) in the cycles, which might occur because of the unique “net”‐like structure of TiO_2_ on the RGO, providing a sufficient amount of interfaces for accepting both electrons and lithium ions simultaneously (Figure 8c). Also, the well‐dispersed sub‐10‐nm TiO_2_ nanoparticles on the RGO are believed to lower the electronic resistance to lithium‐ion diffusion and facilitate lithium transport in the electrode, thus, improving the electrode rate performance and stability.

## 3 Conclusion

In summary, we report a green and simple biomineralization‐inspired method to create “net”‐like interconnected TiO_2_ nanoparticles conformably covering RGO with high loading density. This method uses polyamine (b‐PEI) as the biomineralization agent and linker to manipulate the nucleation, growth, and crystallization of TiO_2_ nanoparticles on graphene oxide. The final obtained anatase TiO_2_/RGO composites demonstrate sub‐10‐nm TiO_2_ nanoparticles with “net”‐like pores, high‐energy (001) facets, ultrathin thickness (10–12 nm), and a high surface area of 172 m^2^ g^−1^. Furthermore, we have shown that the anatase TiO_2_/RGO composites display an excellent rate capability and long cycle life when used as the anode material for lithium ion batteries. A capacity of 155 mAh g^−1^ is retained after 50 cycles at the rate of 5C, and a capacity of 115 mAh g^−1^ is retained after 2000 cycles at the rate of 25C. Such excellent performance is believed to partly originate from the reversible interfacial lithium storage of the material, which is confirmed by discharge curve analysis.

## 4 Experimental Section

*Preparation of Amorphous TiO_2_/GO Composite*: All reagents were purchased commercially and used without purification. In a typical procedure, b‐PEI (25 mL, 0.6 wt%; Sigma–Aldrich, USA) was added to GO (50 mL, 0.5 mg mL^−1^ Graphene Supermarket, USA). After stirring for 8 h to allow b‐PEI to adsorb on GO, the solution was centrifuged (10 000 r min^−1^) and washed with deionized water three times to remove any residual b‐PEI. The resulting GO/b‐PEI composite was dispersed in deionized water (25 mL) and stirred for 20 min, then an aqueous solution of titanium bis(ammonium lactato) dihydroxide (10 mL, 25 wt%, Alfa Aesar, USA) was added dropwise into it to obtain an amorphous TiO_2_ layer on the surface. After being centrifuged and washed with deionized three times, the obtained GO/b‐PEI/Ti−O composites were freeze‐dried and annealed in a reducing atmosphere (5% H_2_/Ar) at 500 °C for 2 h to give anatase TiO_2_/RGO composites.

*Material Characterization*: Field‐emission scanning electron microscopy (FESEM) images were obtained using a Hitachi, S‐4300 (Japan) scanning electron microscope. X‐ray diffraction (XRD) was carried out using a Bruker D8 Advance (Germany) diffractometer with Cu Kα radiation under 40 kV and 40 mA conducted condition, and data were acquired from in 2θ° range of 10°–80° at a rate of 0.02° s^−1^. Transmission electron microscopy (TEM) images were obtained using a FEI Tecnai F20 microscope. Powder samples for TEM were prepared by deposition onto a copper microgrid coated with carbon. Scanning transmission electron microscopy high‐angle annular dark field (STEM‐HAADF) images were obtained using a FEI Tecnai F30 microscope. The atomic force microscopy (AFM) (Cypher, Asylum Research, Santa Barbara, CA) topography image is collected under tapping mode, the scan rate is 2 Hz, the cantilever is Olympus AC240, and resonant frequency is about 70 kHz. Spring constant is about 2 nN nm^−1^. The current image is collected under orca mode, scan rate is 4 Hz, the cantilever is NT‐MDT CONTPt (Cr/PtIr coating), and spring constant is about 0.2 nN nm^−1^. The force applied to the cantilever during the experiment is about 4 nN. Raman spectra were recorded on a Raman spectrometer (LabRAM HR Evolution). Thermogravimetric analysis (TGA) was conducted on a TG‐DTA instrument (Seiko Instruments, 6300), and the sample was heated from 25 °C to 800 °C in air at a rate of 10 °C min^−1^ under the air atmosphere. Nitrogen adsorption and desorption isotherms at 77 K were obtained with a pore‐size analyzer (Autosorb, Quantachrome, USA). X‐ray photoelectron spectroscopy (XPS) was performed on an Axis Ultra (Kratos Analytical, Ltd.) instrument with monochromatized Al Kα radiation and an energy resolution of 0.48 eV. Electron paramagnetic resonance (EPR) spectra were collected at liquid‐nitrogen temperature using an EPR spectrometer (ES‐FA200, JEOL, Japan).

*Electrochemical Measurements*: For half‐cell testing, 2032 coin cells were assembled in an argon‐filled glove box, where both the moisture and oxygen contents were <0.5 ppm. The slurry of electrode material containing 70 wt% TiO_2_, 20 wt% acetylene black, and 10 wt% polyvinylidene fluoride (Aldrich) was pasted onto stainless steel foil with active material loading of 1.0–1.5 mg cm^−2^. Pure lithium foil (Aldrich) was used as the counter electrode, and the electrolyte was 1 m LiPF6 in a 50:50 w/w mixture of ethylene carbonate and diethyl carbonate. Glass fiber (GF/D, Whatman) was used as a separator. A battery tester (Neware, Shenzhen, China) was used to conduct the galvanostatic measurements. Cyclic voltammetry (CV) was measured over the potential range from 3.0 to 1.0 V at a scanning rate of 0.1 mV s^−1^ using a potentiostat (VMP3, Bio Logics, France). The AC impedance of the samples was determined using the same potentiostat, and impedance spectra were obtained by applying a sine wave with amplitude of 5.0 mV over the frequency range from 100 kHz to 0.01 Hz.

## Supporting information

As a service to our authors and readers, this journal provides supporting information supplied by the authors. Such materials are peer reviewed and may be re‐organized for online delivery, but are not copy‐edited or typeset. Technical support issues arising from supporting information (other than missing files) should be addressed to the authors.

Supplementary
