# Supplementary material for: A Biomineralization Strategy for “Net”‐Like Interconnected TiO2 Nanoparticles Conformably Covering Reduced Graphene Oxide with Reversible Interfacial Lithium Storage
Source: Adv Sci (Weinh). 2015 Aug 25;2(11):1500176. doi: 10.1002/advs.201500176 (PMC5049643; doi:10.1002/advs.201500176)
Supplement: Supplementary file 1 — Supplementary [file ADVS-2-0h-s001.pdf]

Copyright WILEY-VCH Verlag GmbH & Co. KGaA, 69469 Weinheim, Germany, 2015.

ADVANCED SCIENCE
Open Access

Supporting Information

for *Adv. Sci.*, DOI: 10.1002/advs. 201500176

A Biomineralization Strategy for "Net"-Like Interconnected TiO_2 Nanoparticles Conformably Covering Reduced Graphene Oxide with Reversible Interfacial Lithium Storage

*Qiang Zhang, Yong Yan, and Ge Chen**

Copyright WILEY-VCH Verlag GmbH & Co. KGaA, 69469 Weinheim, Germany, 2013.

Supporting Information

Title

Bio-mineralization Strategy toward "Net"-like Interconnected TiO_2 Nanoparticles Conformably Covering Reduced Graphene Oxide with Reversible Interfacial Lithium Storage

*Qiang Zhang, Yong Yan‡ and Ge Chen**

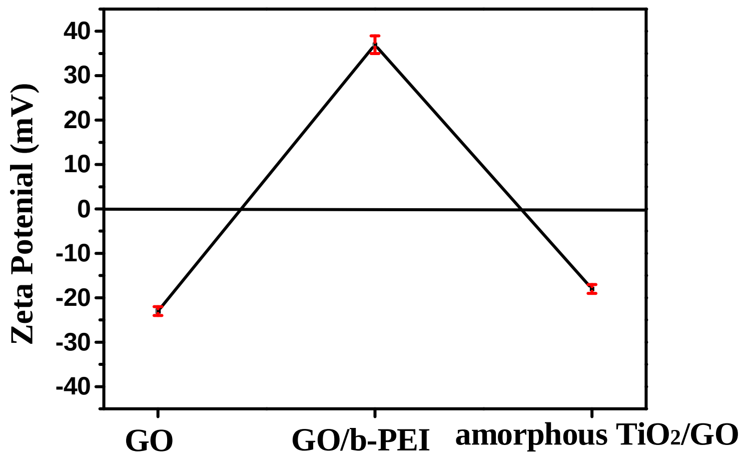

Figure S1. Zeta potentials of GO, GO/b-PEI, and amorphous TiO_2/GO composites.

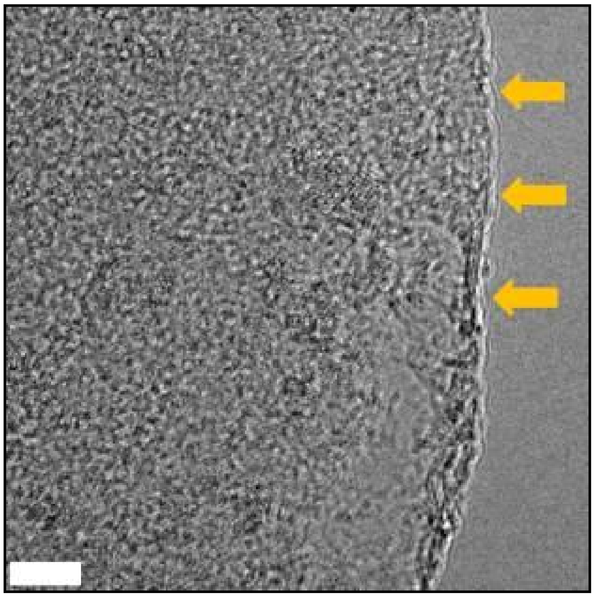

Figure S2. HRTEM image of amorphous TiO_2/GO composite; the edge of GO marked by the orange arrow indicates the ultrathin nature (3–4 layers), bar 5 nm.

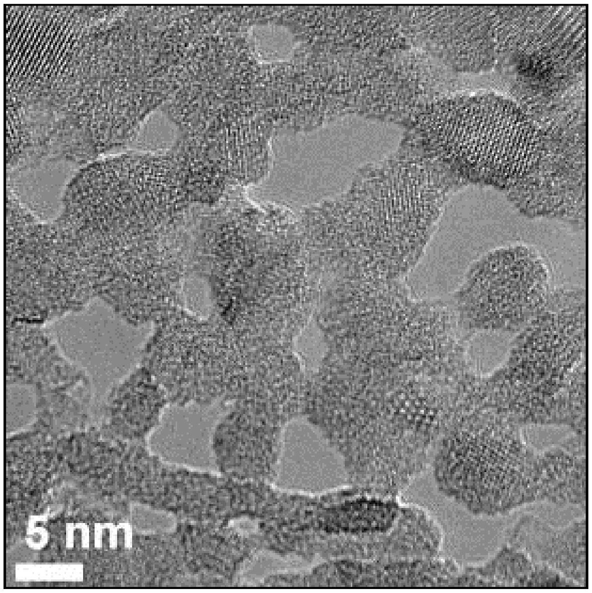

Figure S3. HRTEM image of anatase TiO_2/RGO composite, indicating the “net”-like structure of TiO_2 conformably covering RGO, bar 5 nm.

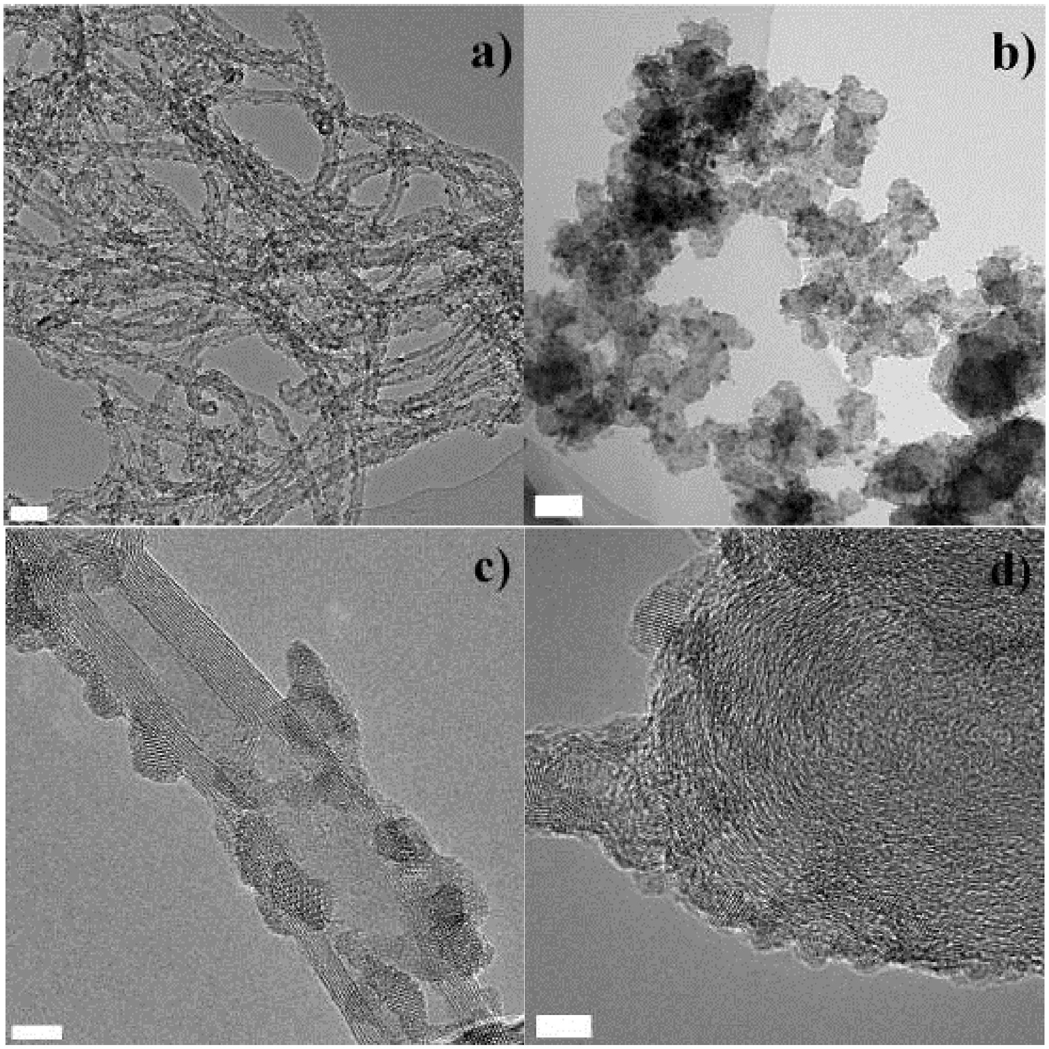

Figure S4. a) TEM image of anatase TiO_2/CNTs, bar 50 nm. b) TEM image of anatase TiO_2/carbon spheres (XC-72), bar 50 nm. c) HRTEM image of anatase TiO_2/CNTs, bar 5 nm. d) HRTEM image of anatase TiO_2/carbon spheres (XC-72), bar 5 nm.

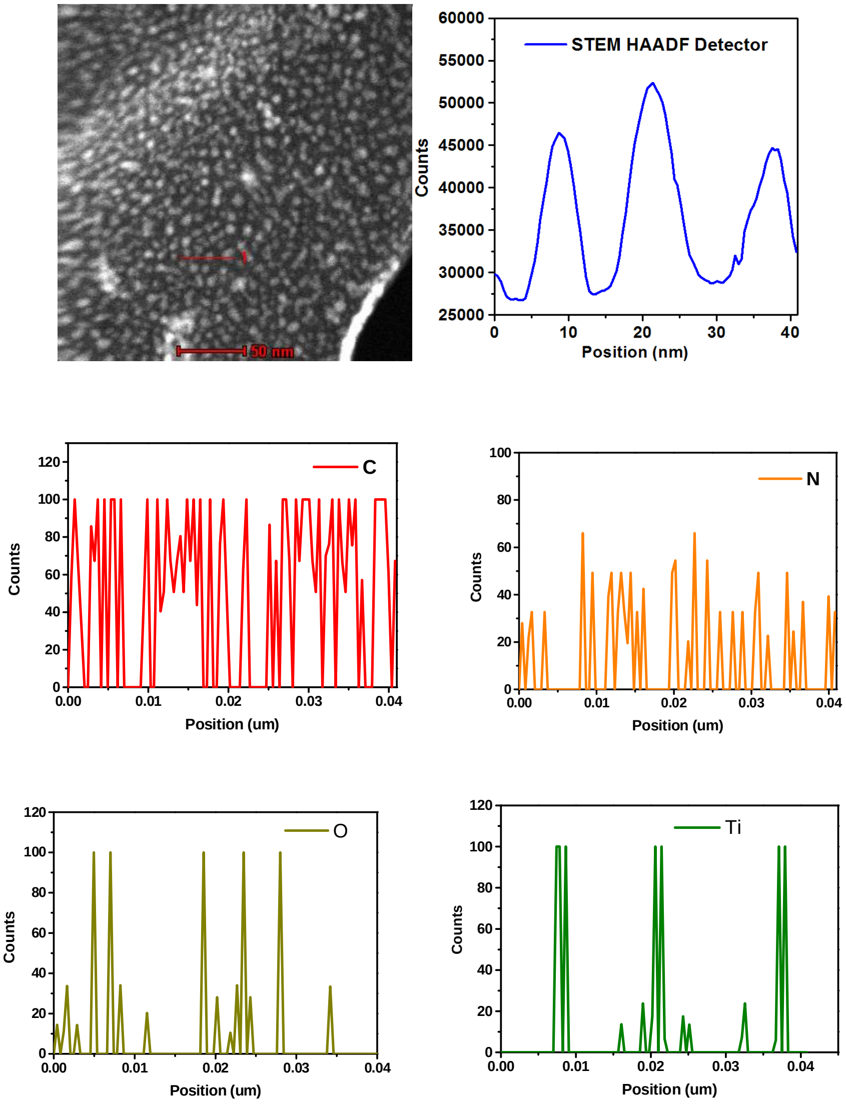

Figure S5. Energy-dispersive X-ray spectroscopy (EDS) line scan analysis based on the STEM-HADDF model demonstrating the distribution of C, N, O, and Ti species; while the Ti and O species are dispersed on the particles, the C and N species are dispersed along the line.

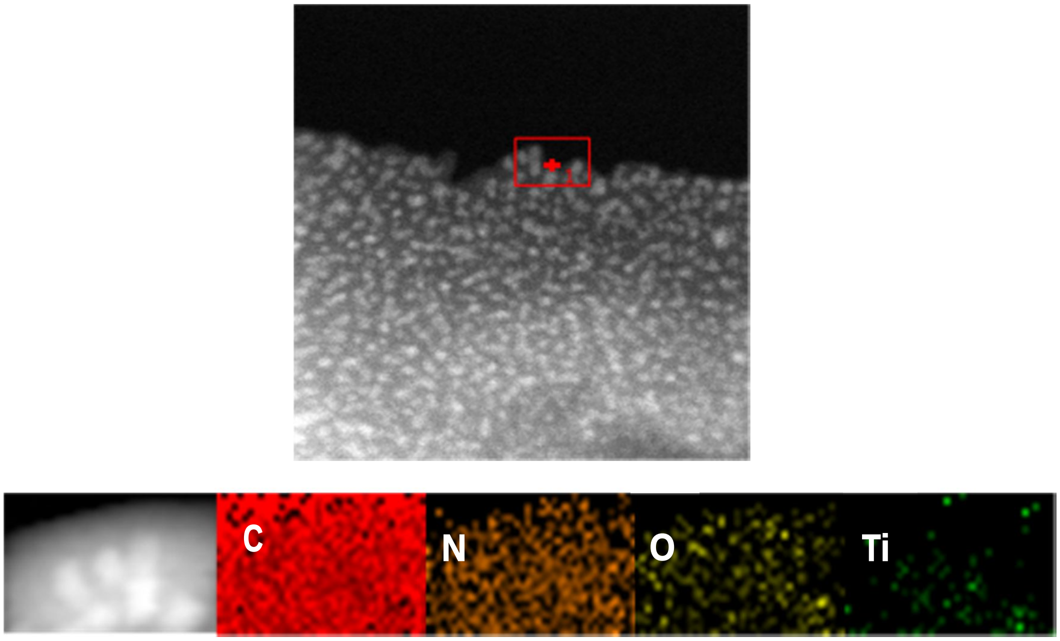

Figure S6. EDS elemental mapping of C, N, O, and Ti species for the anatase TiO_2/RGO composite.

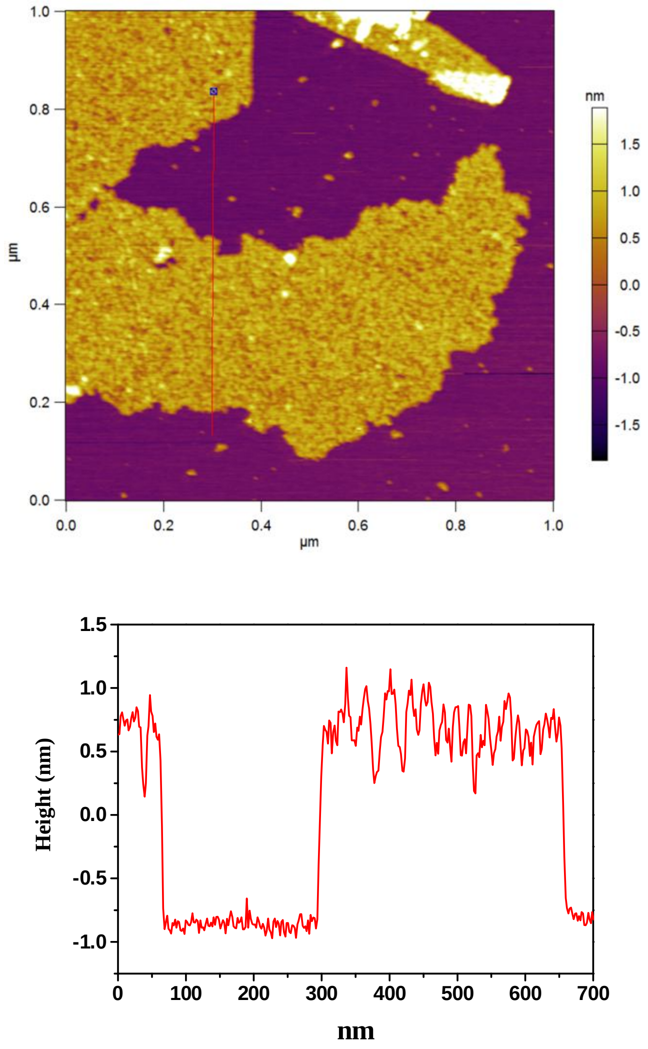

Figure S7. Topography image of GO on a mica substrate and the corresponding height-profile analysis along the line.

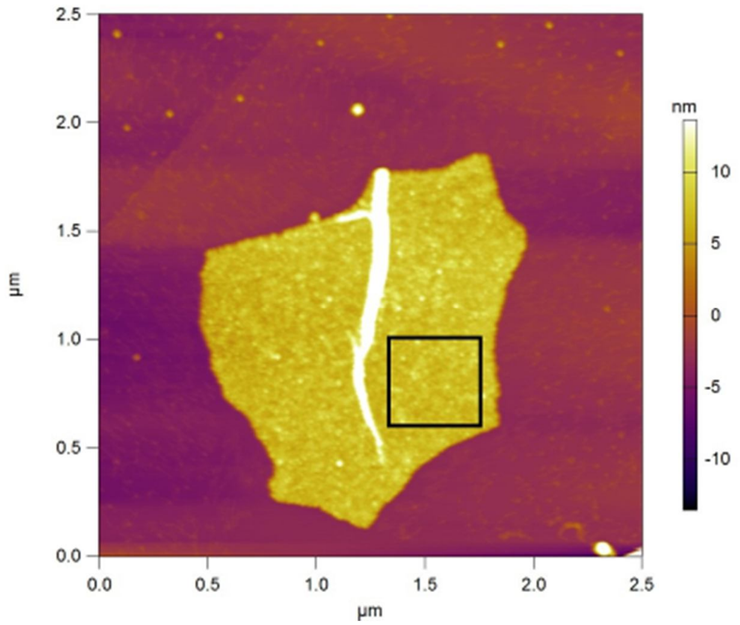

Figure S8. Topography image of anatase TiO_2/RGO on the HOPG substrate; the black square region is ready for current image collecting.

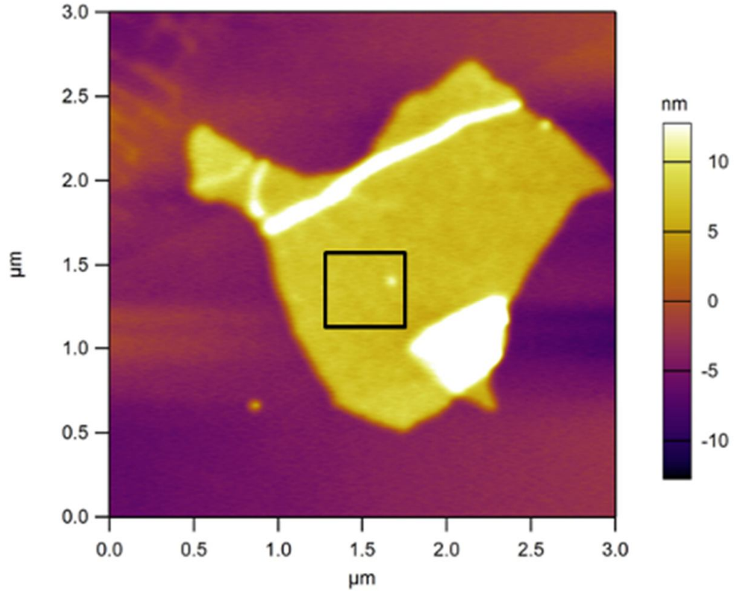

Figure S9. Topography image of amorphous TiO_2/GO on the HOPG substrate; the black square region is ready for current image collecting.

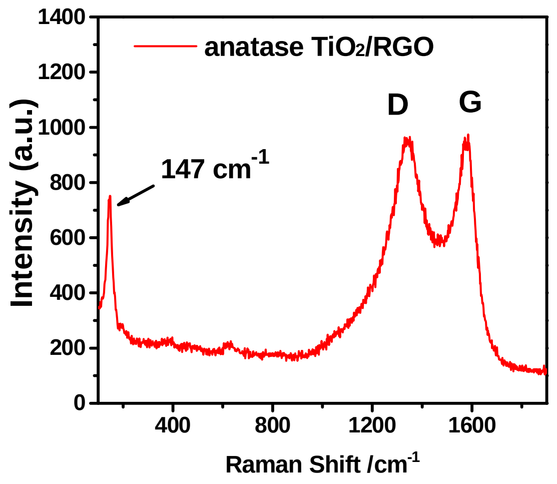

Figure S10. Raman spectrum of the anatase TiO_2/RGO composite.

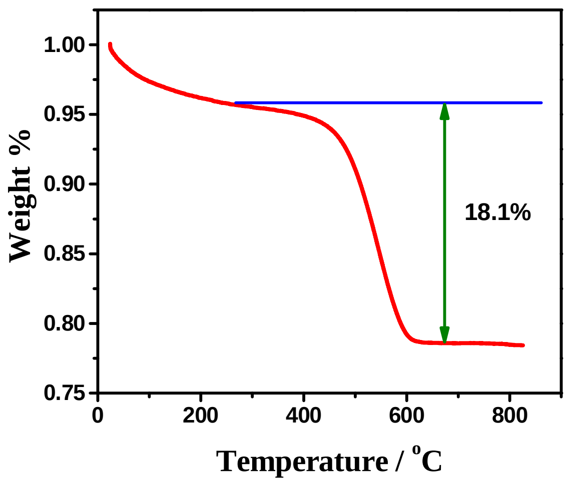

Figure S11. TGA analysis of the anatase TiO_2/RGO composite in air.

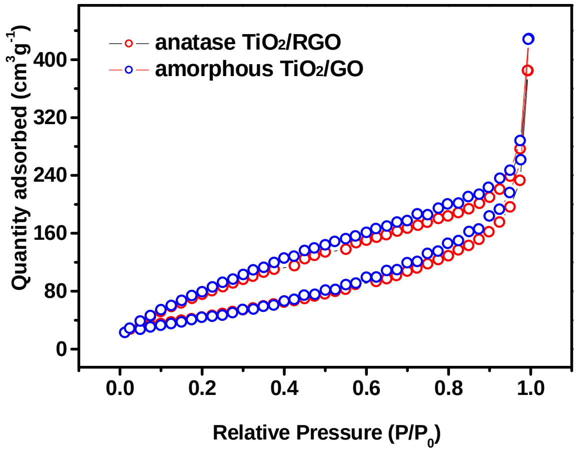

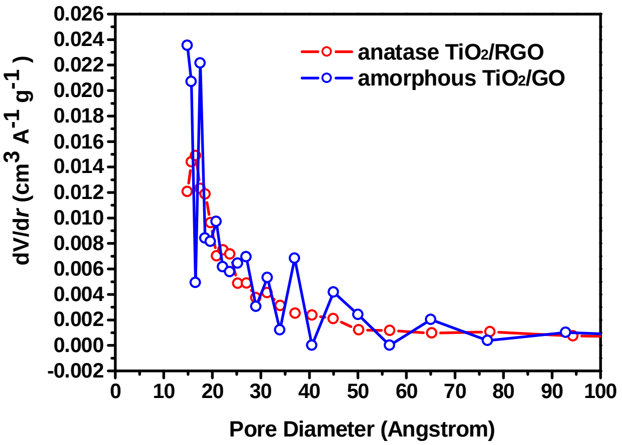

Figure S12. N_2 adsorption/desorption isotherms of amorphous TiO_2/GO and anatase TiO_2/RGO composites and corresponding pore size distribution results analyzed by the BJH method.

Table S1 Comparison of electrochemical performance of obtained anatase TiO_2/RGO with other relative anodes materials.

Materials	Discharge density	Capacity (mAh g^{-1})	Cycle numbers	Reference
Anatase TiO_2/RGO	5C	151	50	Current study
Anatase TiO_2-FGS	1C	160	100	[9]
TiO_2-RGO	5C	175	30	[11]
TiO_2-RGO	5.9C	143	35	[16]
TiO_2/GAs	5.9C	130	40	[18]
Graphene-TiO_2	5C	130	100	[10]
TiO_2-RGO	5C	152	100	[71]
TiO_2 −GNS –CNT	10C	111	100	[72]
Graphene-TiO_2-C	0.59C	95	20	[73]
